# Real-world experience with secukinumab in the entire axial spondyloarthritis spectrum

**DOI:** 10.3389/fmed.2023.1156557

**Published:** 2023-05-11

**Authors:** Francisca Sivera, Victoria Núñez-Monje, Cristina Campos-Fernández, Isabel Balaguer-Trull, Montserrat Robustillo-Villarino, Marta Aguilar-Zamora, Marta Garijo-Bufort, Juan Miguel López-Gómez, Carolina Peña-González, Isabel de la Morena, Diego Bedoya-Sanchís, Liliya Yankova-Komsalova, Arantxa Conesa-Mateos, Anna Martínez-Cristóbal, Francisco Javier Navarro-Blasco, José Miguel Senabre-Gallego, Juan José Alegre-Sancho

**Affiliations:** ^1^Rheumatology Department, Hospital General Universitario de Elda, Alicante, Spain; ^2^Departament of Clinical Medicine, Universidad Miguel Hernandez, Elche, Spain; ^3^Rheumatology Department, Hospital Universitario Dr Peset, Valencia, Spain; ^4^Rheumatology Department, Hospital General Universitario, Valencia, Spain; ^5^Reumatology Unit, Internal Medicine Department, Hospital Universitario de la Plana, Villarreal, Spain; ^6^Rheumatology Department, Hospital de Sagunto, Sagunto, Spain; ^7^Rheumatology Department, Hospital Francesc de Borja, Gandía, Spain; ^8^Rheumatology Department, Hospital Clínico Universitario de Valencia, Valencia, Spain; ^9^Rheumatology Department, Hospital Marina Salud, Denia, Alicante, Spain; ^10^Rheumatology Department, Hospital General Universitari de Castelló, Castellón, Spain; ^11^Rheumatology Department, Hospital Universitario de La Ribera, Alzira, Spain; ^12^Rheumatology Department, Hospital Universitario de Elche, Elche, Alicante, Spain; ^13^Rheumatology Department, Hospital Marina Baixa, La Vila Joiosa, Spain

**Keywords:** secukinumab, effectiveness, axial spondyloarthritis, non-radiographic axial spondyloarthritis, ankylosing spondylitis, real-world evidence

## Abstract

**Background:**

Secukinumab is a biologic disease-modifying antirheumatic drug (bDMARD) that has demonstrated efficacy in the treatment of axial spondyloarthritis (axSpA, i.e., ankylosing spondylitis and non-radiographic axSpA) across various clinical trials. However, data of secukinumab in clinical practice is still limited. Here, we aimed to provide real-world data on secukinumab use, effectiveness, and persistence in axSpA.

**Patients and methods:**

Retrospective, multicenter study of patients with a diagnosis of axSpA treated with secukinumab at 12 centers up to June 2021 in the Valencian Community (Spain). Information was gathered on BASDAI measurement, pain, patient and physician global assessment (ptGA, phGA) using a 100-mm visual analog scale (VAS), persistence and other secondary variables by treatment line (first, second, and ≥ third) for up to 24 months.

**Results:**

221 patients were included (69% men; mean age [standard deviation, SD]: 46.7 [12.1] years old). Secukinumab was used as a first-line bDMARD in 38% of patients, as a second-line in 34% and as a ≥ hird-line in 28%. The percentage of patients achieving low disease activity (BASDAI<4) increased from 9% at baseline to 48% at month 6 and was maintained (49%) up to month 24. Improvements in BASDAI were observed across all treatment lines: in naïve patients (month 6: −2.6; month 24: −2.7), in second-line (month 6: −1.9; month 24: −3.1), and in patients on third lines (month 6: −1.3; month 24: −1.7). Reductions in mean pain VAS (−23.3; −31.9), ptGA (−25.1; −31.9) and phGA (−25.1; −31) were also observed at 6 and 24 months. Secukinumab showed an overall 12-months persistence rate of 70% (95% confidence interval [CI]: 63–77%) and a 24-months persistence rate of 58% (95% CI, 51–66%). Patients receiving first-line secukinumab had the highest 24-months persistence rate (*p* = 0.05).

**Conclusion:**

Secukinumab improved disease activity in axSpA patients, especially in naive, and second-line patients, which was accompanied by high persistence rates up to 24 months.

## 1. Introduction

Axial spondyloarthritis (axSpA) is a chronic, inflammatory, rheumatic disease that affects the axial skeleton, usually starting in the third decade of life (1). AxSpA can be classified into classic ankylosing spondylitis (AS) or non-radiographic axSpA (nr-axSpA), based on the respective presence or absence of sacroiliitis on conventional radiographs (2). Approximately 10%–40% of nr-axSpA patients progress to AS over a period of 2–10 years (3); and nr-axSpA and AS are predominantly considered as part of the same disease spectrum (4). Signs of active inflammation, such as elevated C-reactive protein (CRP) levels or evidence of sacroiliitis on magnetic resonance imaging (MRI) predispose to a more rapid progression of structural damage and can predict conversion of nr-axSpA to AS (3).

Axial pain, stiffness, and fatigue are among the most common complains in axSpA. Patients can also present peripheral manifestations (arthritis, enthesitis, or dactylitis), extra-musculoskeletal manifestations (anterior uveitis or psoriasis) and significant comorbidities, including cardiovascular disease and depression (1, 5). These symptoms profoundly impact patients’ health-related quality of life (HRQoL), and affect their professional, social, and family roles (6). The impact on HRQoL is independent of the radiographic status, as both AS and nr-axSpA patients show a similar degree of impairment (7).

The Assessment of SpondyloArthritis international Society (ASAS) and the European Alliance of Associations for Rheumatology (EULAR) recommend the use of biologic disease-modifying antirheumatic drugs (bDMARDs) in patients with persistently high disease activity despite conventional treatments (8). IL-17 inhibitors (IL-17i) or tumor necrosis factor inhibitors (TNFi) are now recommended as first-line bDMARD (8). Given that up to 50% of patients treated with TNFi do not achieve a clinically significant response (9), IL-17i have acquired a key role in axSpA treatment. Secukinumab, an IL-17Ai monoclonal antibody, has demonstrated efficacy and safety in the treatment of AS (10–16) and nr-axSpA (17), leading to its approval in the EU (in 2015 for AS and in 2020 for nr-axSpA). So far, more than 875,000 patients have been treated with secukinumab across its four approved therapeutic indications worldwide (data on file).

Randomized clinical trials (RCT) have showed the benefit of secukinumab in reducing signs and symptoms in patients with axSpA who were either bDMARD-naive or had a history of treatment with TNFi (11, 17, 18). However, real-world evidence (RWE) on secukinumab effectiveness and persistence in patients with axSpA is still limited (19–21). Establishing secukinumab effectiveness and retention rates based on prior bDMARD is key for improving treatment decision-making. This study aims to describe the use of secukinumab in patients with axSpA in routine clinical practice and assesses its effectiveness and persistence per bDMARD treatment line.

## 2. Methods

### 2.1. Study design and population

This was a non-interventional, retrospective, and multicenter study in patients with axSpA treated with secukinumab at 12 centers in the Valencian Community (Spain). All adult patients with a diagnosis of axSpA as per their treating physician and who were receiving or had received treatment with secukinumab up to June 2021, were included in the study. Patients were excluded if they had received secukinumab in the context of a clinical trial or as off-label therapy. The study was approved by the Ethics Committee of the General University Hospital in Elda (Alicante). Written informed consent was not required for this study in accordance with the national legislation (Real Decreto 957/2020). The study was conducted in accordance with the ethical principles of the Declaration of Helsinki, Good Clinical Practice (GCP), and in compliance with European and local requirements.

### 2.2. Study endpoints

The primary endpoint was the percentage of patients who started secukinumab on each treatment line (first, second, or ≥ third bDMARD line) and with either 150 mg or 300 mg in clinical practice. Secondary endpoints of effectiveness were assessed by BASDAI (Bath Ankylosing Spondylitis Disease Activity Index; from 0 to 10, with higher scores indicating higher disease activity), presence of enthesitis and dactylitis, skin involvement, C reactive protein (CRP, mg/L), and pain, patient’s global assessment (ptGA) and physician’s global assessment (phGA) of the disease measured by a visual analogue scale (VAS; from 0 to 100, with higher scores indicating worse pain/disease activity) from baseline to 24 months after secukinumab initiation. The incidence of uveitis, secukinumab persistence at 24 months and reasons for discontinuation were also collected. All secondary endpoints are showed in all patients and per treatment line (first, second, or ≥ third bDMARD line).

### 2.3. Data analysis

Measures of central tendency and dispersion were presented [mean (standard deviation; SD) for continuous variables and frequencies and percentages for categorical variables] for data on patient characteristics and secukinumab effectiveness; 95% confidence intervals (CI) were calculated for secukinumab use and persistence. Percentages reported in the Results section have been rounded up (if the tenths digit ≥5) or down (if <5).

The incidence rate of uveitis was calculated as the ratio of the number of patients with uveitis during secukinumab treatment to the sum of secukinumab treatment periods (time from initiation of secukinumab treatment to discontinuation or data collection) for all patients during the period of data collection.

Secukinumab persistence was analyzed by using the Kaplan Meier method. The persistence of secukinumab was quantified as the time from the start of secukinumab until the end of treatment (definitive discontinuation) or until the end of data collection in those patients who continued on treatment. Differences between groups (naïve, second line, third or posterior lines) in secukinumab persistence were analyzed using the log rank test.

## 3. Results

### 3.1. Patient profile

A total of 221 patients diagnosed with axSpA met the selection criteria and were included in the study. Table 1 shows the demographic and clinical characteristics of these patients and Supplementary Table S1 displays this information based on diagnosis of AS or nr-axSpA. Briefly, mean (SD) age was 46.7 (12.1) years old and 152 (69%) patients were men. The mean (SD) delay from first symptoms to axSpA diagnosis was 4.2 (6) years. Seventy-four percent of patients were HLA-B27 positive. The percentage of patients with at least one comorbidity was 45%. Regarding baseline disease activity, the mean (SD) scores were: BASDAI 6.4 (1.8), pain VAS 66.7 (23.4), ptGA 64.9 (24.3), and phGA 55.1 (22.7).

**Table 1:** Patient baseline demographic and clinical characteristics.

	axSpA (*n* = 221)
Gender (male), *n* (%) [*N*]	152 (69) [221]
Ethnicity (Caucasian), *n* (%) [*N*]	205 (93) [221]
Age, mean (SD) [*N*]	46.7 12.1 [221]
BMI (kg/m^2^), mean (SD) [*N*]	27.4 (5.3) [127]
Obese (BMI ≥30), *n* (%) [*N*]	40 (31.5) [127]
Current smokers, *n* (%) [*N*]	64 (35) [182]
Age at symptoms initiation, mean (SD) [*N*]	35.7 (11.7) [179]
Age at diagnosis, mean (SD) [*N*]	39.4 (11.7) [210]
Years from first symptoms to diagnosis, mean (SD) [*N*]	4.2 (6) [179]
Nr-axSpA, *n* (%) [*N*]	39 (17.7) [220]
Peripheral arthritis, *n* (%) [*N*]	62 (28) [221]
Enthesitis (yes), *n* (%) [*N*]	30 (17) [174]
Number at baseline assessment, mean (SD) [*N*]	2.5 (1.8) [26]
Dactylitis (yes), *n* (%) [*N*]	2 (1) [176]
Skin manifestation (yes), *n* (%) [*N*]	12 (9) [202]
Nail manifestation (yes), *n* (%) [*N*]	2 (1) [202]
Uveitis (yes), *n* (%) [*N*]	12 (6) [203]
Prior number of flares, mean (SD) [*N*]	2 (0.9) [10]
BASDAI, mean (SD) [*N*]	6.4 (1.8) [170]
Pain VAS, mean (SD) [*N*]	66.7 (23.4) [139]
PtGA, mean (SD) [*N*]	64.9 (24.3) [101]
PhGA, mean (SD) [*N*]	55.1 (22.7) [89]
CRP (mg/dL), mean (SD) [*N*]	6.6 (13) [207]
ESR (mm/h), mean (SD) [*N*]	20.8 (20.5) [199]
HLA-B27 (positive), *n* (%) [*N*]	156 (73) [212]
Prior anti-TNFα, *n* (%) [*N*]	137 (80) [171]
Comorbidities (any), *n* (%) [*N*]	100 (45) [221]
Hypertension (yes), *n* (%) [*N*]	50 (50) [100]
Dyslipidemia (yes), *n* (%) [*N*]	38 (38) [100]
Depression (yes), *n* (%) [*N*]	28 (28) [100]
Diabetes (yes), *n* (%) [*N*]	12 (12) [100]
Tuberculosis/latent tuberculosis infection (yes), *n* (%) [*N*]	24 (24) [100]
Fatty liver disease (yes), *n* (%) [*N*]	13 (13) [100]
Cardiovascular events (yes), *n* (%) [*N*]	10 (10) [100]
Neoplasms (yes), *n* (%) [*N*]	11 (11) [100]

Valid percentages (i.e., percentage of patients with data in the variable) are showed here. AS, ankylosing spondylitis; BASDAI, Bath Ankylosing Spondylitis Disease Activity Index; BMI body mass index; CRP, C-Reactive Protein; ESR, Erythrocyte sedimentation rate; HLA, Human leukocyte antigen; *N*, total number of patients in the study population; ptGA, patient global assessment; phGA, physician global assessment; SD, standard deviation.

Most patients (77%) had received at least one DMARD prior to starting secukinumab (62% had received a bDMARD, and 48% a csDMARD) (Supplementary Table S2). Secukinumab was initiated a mean (SD) of 11 (9) years after first symptoms.

### 3.2. Secukinumab Use

**Figure 1:**
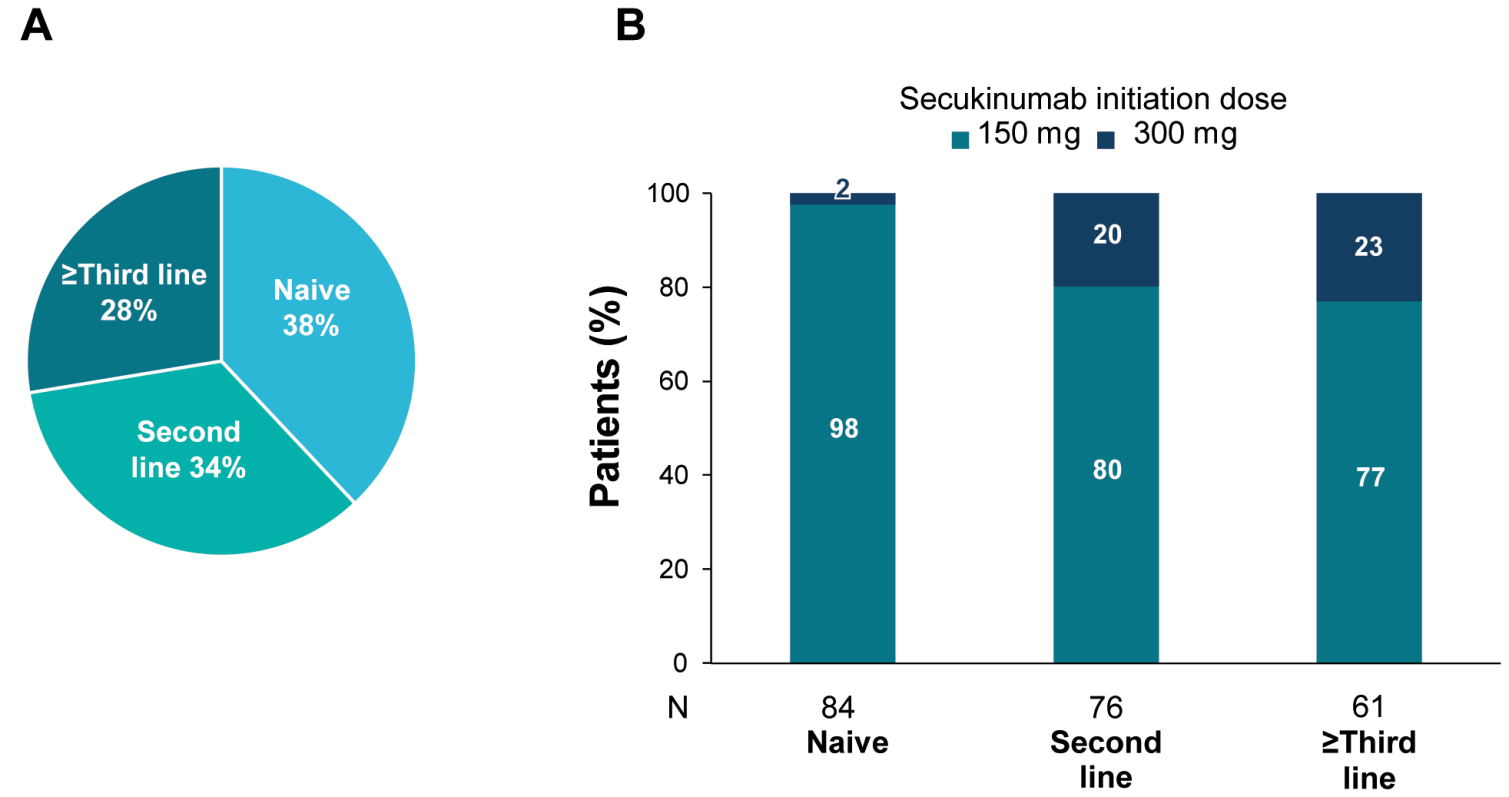
Secukinumab use by bDMARD treatment line.

As shown in Figure 1A, 38% (95% CI: 32–45%) of the patients received secukinumab as a first-line bDMARD (bDMARD-naive), 34% (95% CI: 28–41%) as a second-line, and 28% (95% CI: 22–34%) as a third or subsequent line. Most (86%) patients initiated treatment with the 150 mg dose; nonetheless, 20% and 23% of bDMARD-experienced patients initiated treatment with the 300 mg dose when secukinumab was initiated as second-line and as third or subsequent line, respectively (Figure 1B). Uptitration to 300 mg during the course of treatment occurred in 19% of patients initiating treatment with secukinumab 150 mg. Doses were increased to 300 mg in 15%, 20%, and 28% of naive, second-line, and third or subsequent-line patients, respectively. Also, most patients initiated treatment in monotherapy (79%) (Table 2). Patients were treated with secukinumab a mean (SD) of 7.3 (7.5) years after diagnosis. Mean (SD) duration of secukinumab treatment was 20.9 (15.5) months (Table 2).

**Table 2:** Secukinumab treatment.

	axSpA (*n* = 221)
Years from first symptoms to secukinumab initiation, mean (SD) [*N*]	11 (9) [179]
Years from diagnosis to secukinumab initiation, mean (SD) [*N*]	7.3 (7.5) [210]
Initial dose: 150 mg, *n* (%) [*N*]	190 (86) [221]
Monotherapy, *n* (%) [*N*]	174 (79) [221]
Duration of secukinumab (months), mean (SD) [*N*]	20.9 (16) [221]

### 3.3. Effectiveness

#### 3.3.1. All patients

**Figure 2:**
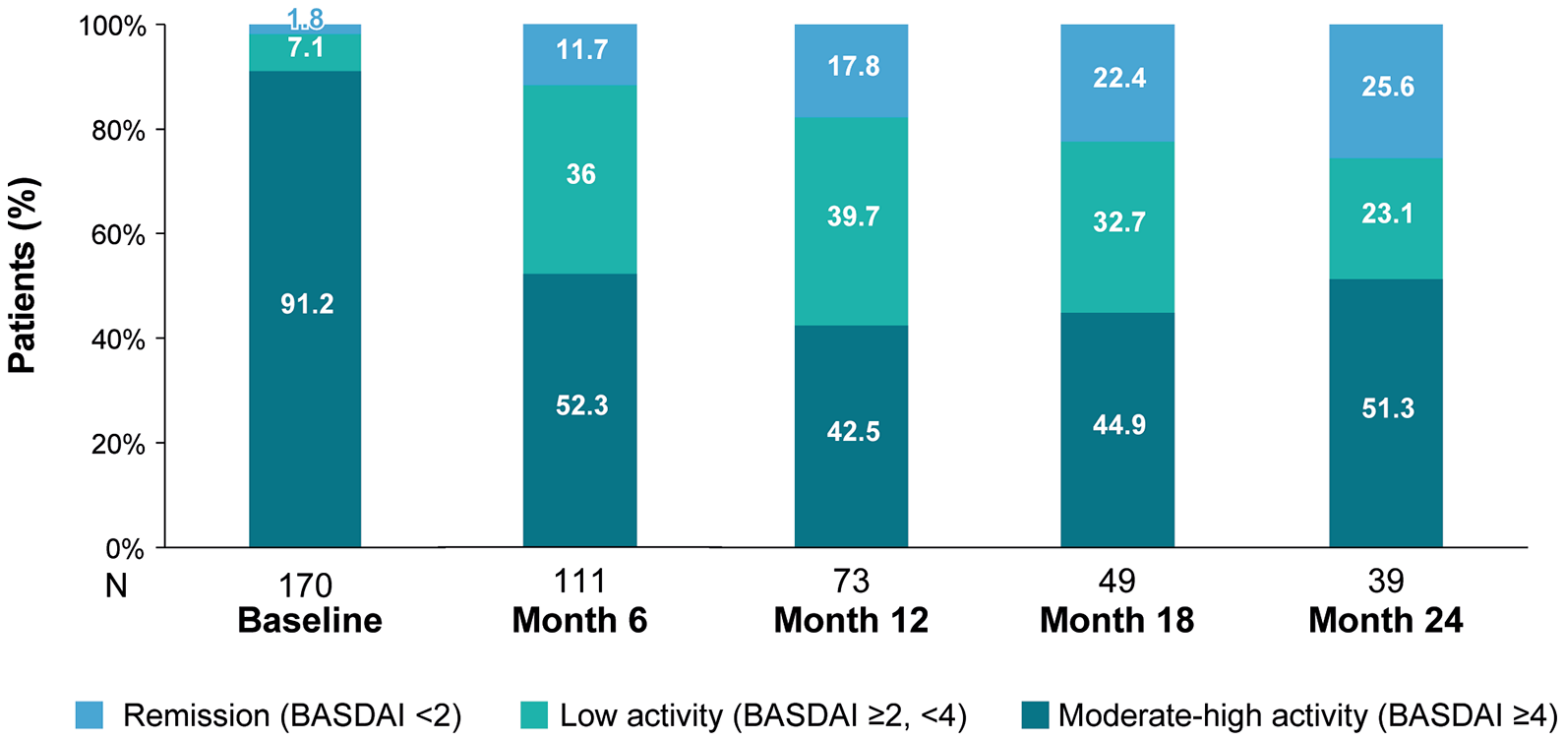
BASDAI disease activity categories during secukinumab treatment.

Under secukinumab treatment, disease activity improved. Mean (SD) BASDAI scores decreased from 6.4 (1.8) at baseline (*n* = 170) to 4.3 (2.2) after 6 months (*n* = 111) and to 3.9 (2.3) after 24 months of follow-up (*n* = 39) (Supplementary Table S3). The percentage of patients achieving low disease activity (BASDAI<4) increased from 9% at baseline to 48% at month 6 and was maintained up to month 24 (48%). Moreover, a quarter (26%) of patients achieved remission (BASDAI<2) by month 24 (Figure 2). Conversely, the percentage of patients with high disease activity (BASDAI≥4) at baseline (91%) decreased at month 6 (52%) and was maintained at 24 months (51%).

**Figure 3:**
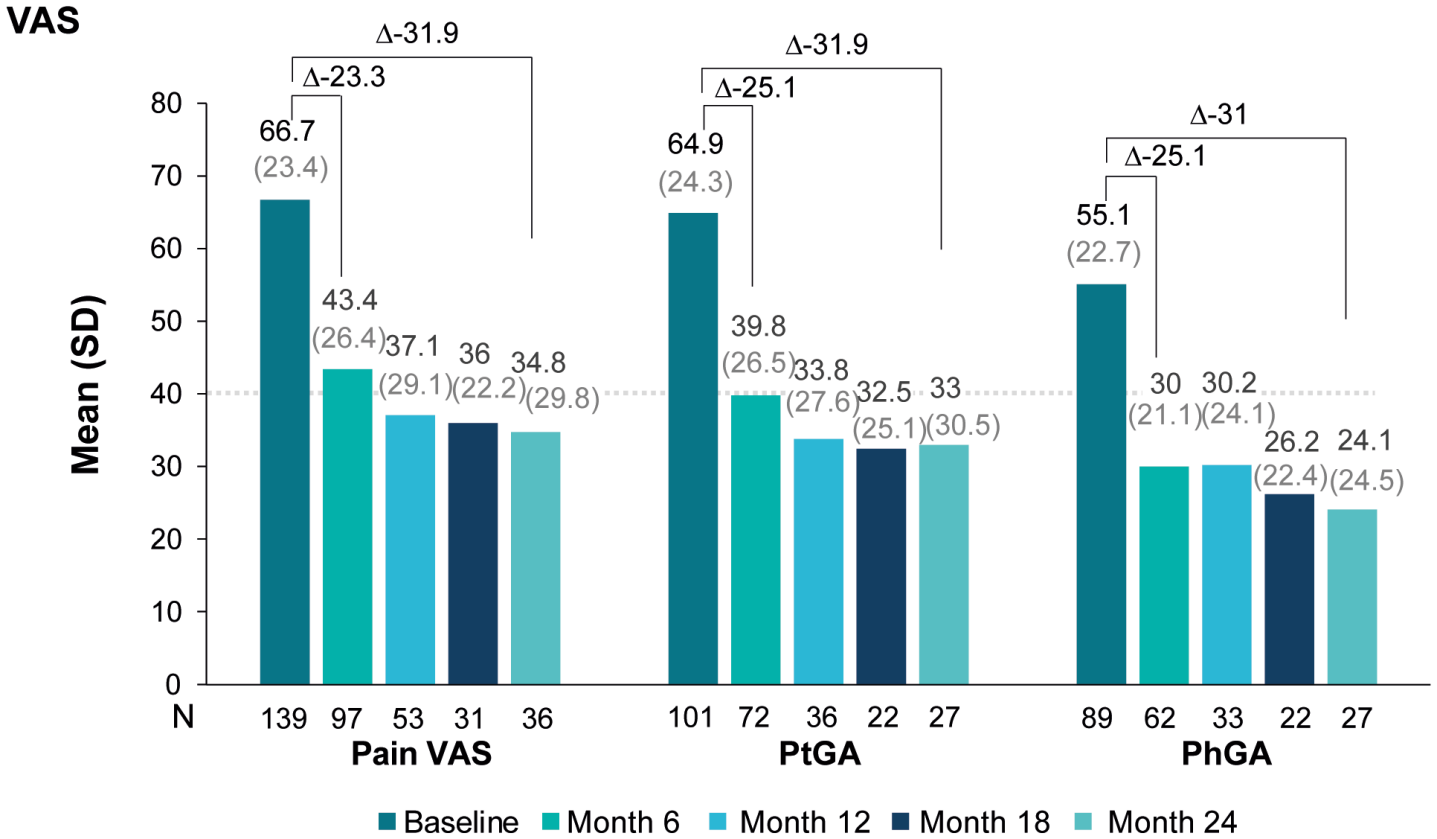
Pain VAS, ptGA, and phGA scores during secukinumab treatment.

Additional assessments such as pain VAS, ptGA and phGA, the presence of enthesitis and CRP also showed similar improvements (Figure 3). At 6 months, mean pain scores improved by 23 points and mean ptGA and phGA by 25 points; the improvements were sustained at 24 months.

The number of patients with at least one enthesitis gradually decreased during secukinumab treatment (17% at baseline, 12% at month 6, and 7% at month 24). Meanwhile, the number of patients with dactylitis remained low and stable, with less than 2% of patients ever presenting dactylitis (0.7% at month 6, 0% at month 12, 1.5% at month 18, and 1.8% at month 24). The percentage of patients without skin manifestations steadily increased from baseline to month 24 months, where 100% of patients had no skin manifestation. The mean (SD) CRP levels decreased from baseline (6.6 [13] mg/L; n = 207) to month 6 (5.0 [10.0] mg/L; n = 161) and remained low at month 24 (4.8 [13.0] mg/L, n = 58). The incidence rate of uveitis was 1.75 per 100 patient-years.

#### 3.3.2. Per treatment line

**Figure 4:**
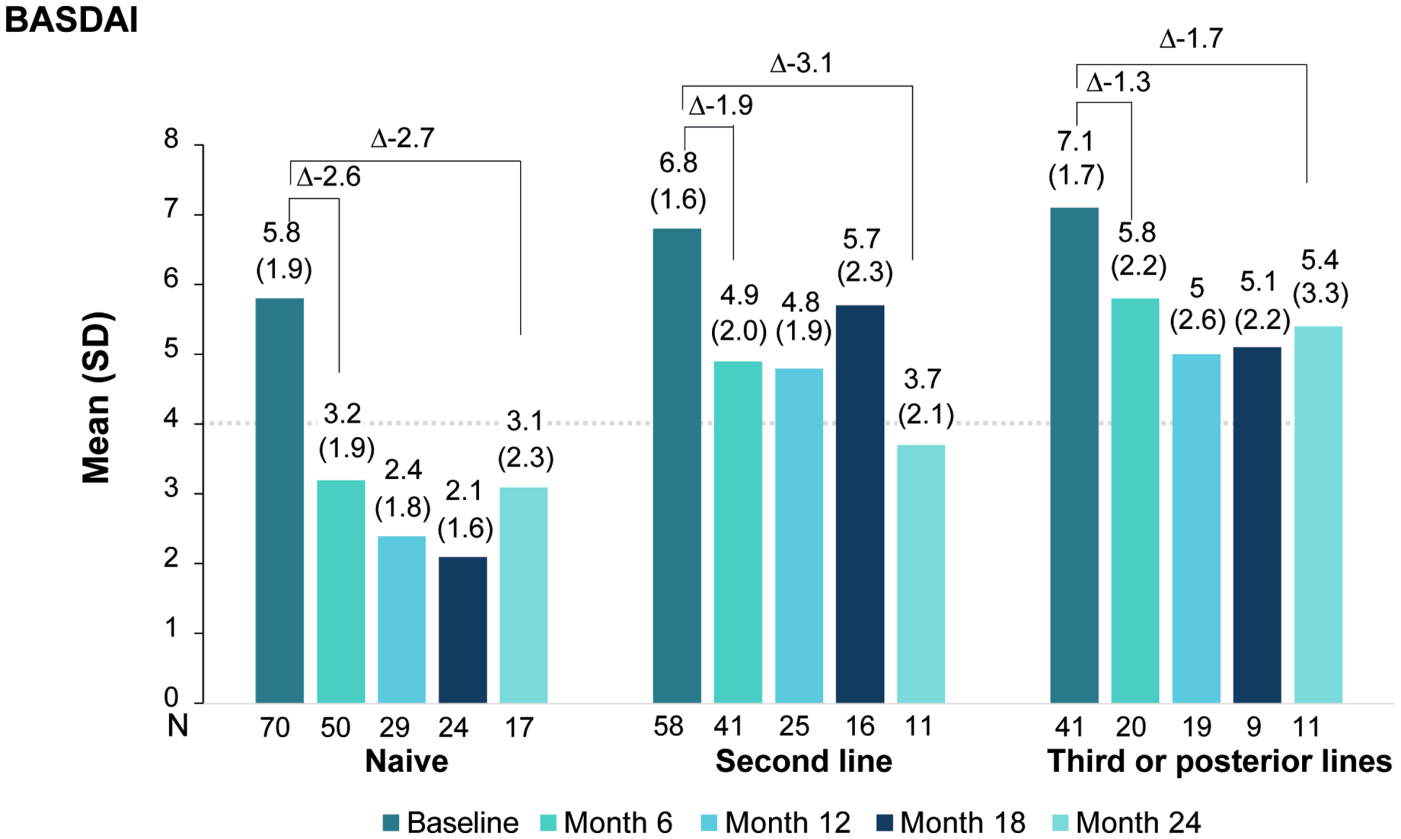
BASDAI score evolution under secukinumab treatment by line of treatment.

As shown in Figure 4, even though mean BASDAI improved across all lines of treatment, naïve patients and second-line patients showed the greatest BASDAI improvement, whereas patients on third or subsequent lines of therapy showed the least improvement. In first line therapy patients, BASDAI decreased 2.6 points at month 6. By month 24, mean BASDAI were under 4 for naïve and second-line, but not for subsequent lines of therapy.

**Figure 5:**
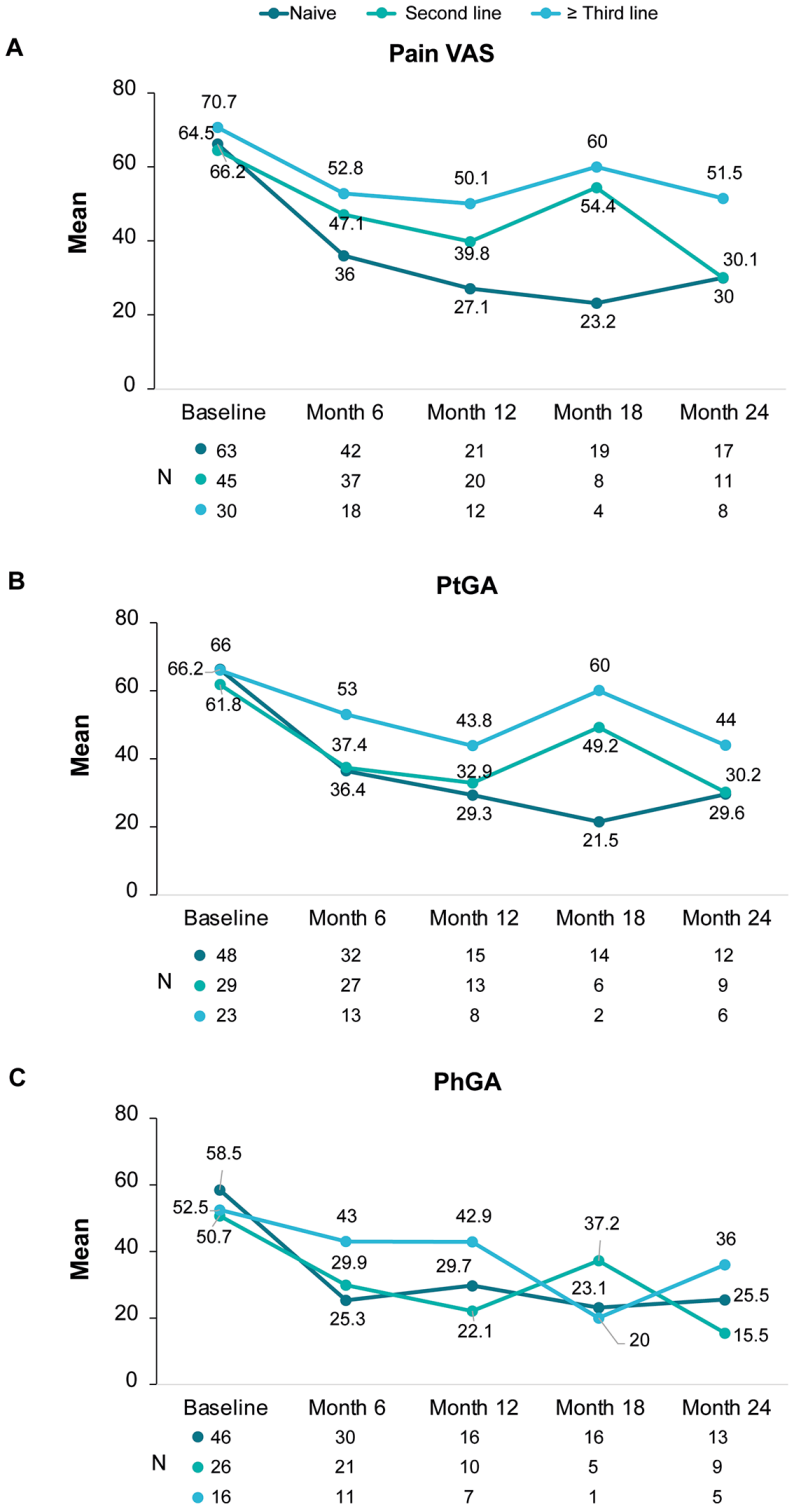
Changes in pain VAS, ptGA and phGA scores under secukinumab treatment according to line of treatment.

Changes in pain VAS, ptGA, and phGA according to treatment line are shown in Figure 5 and Supplementary Table S4. Overall, VAS scores improved to a higher extent in naive patients than in patients previously treated with another bDMARD. Nonetheless, every group showed clear improvements from baseline to month 6 that were maintained or further improved up to month 24.

### 3.4. Persistence

**Figure 6:**
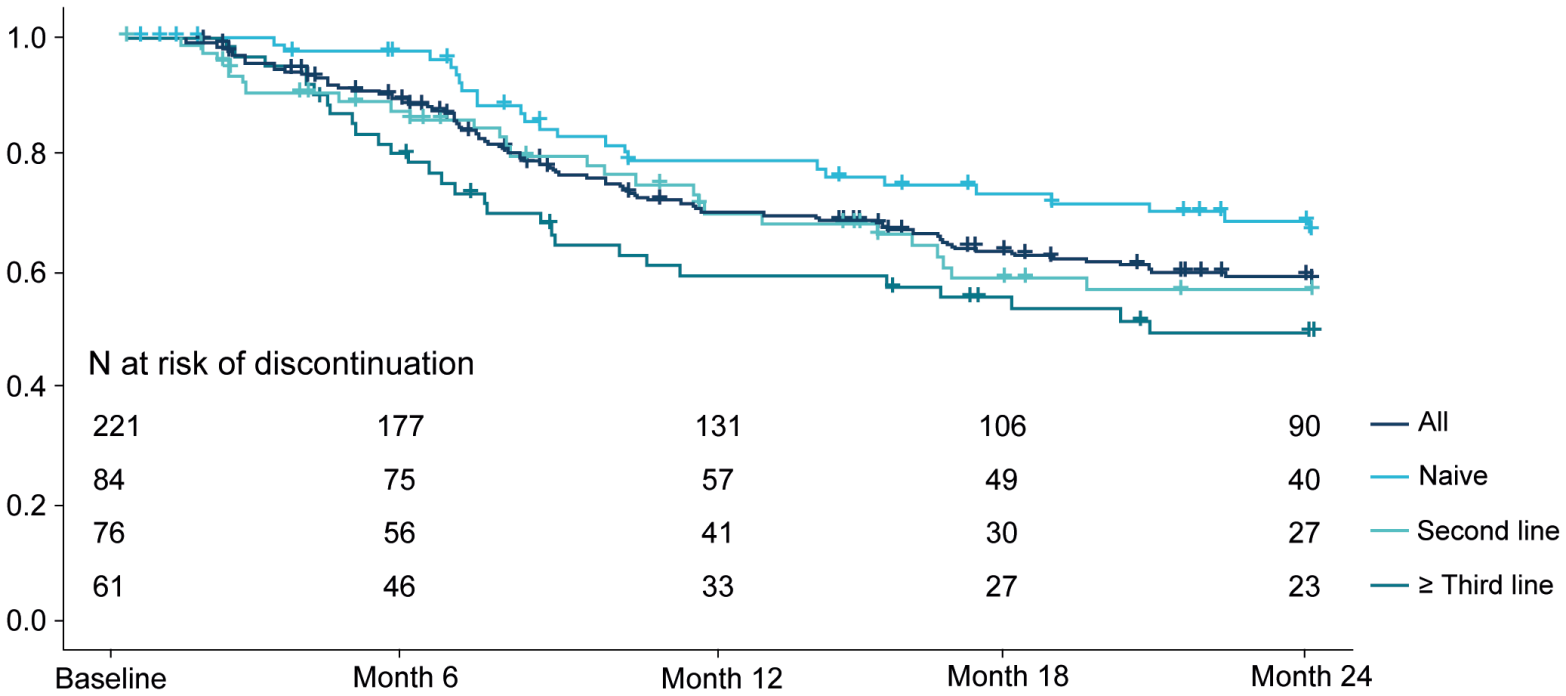
Secukinumab persistence per treatment line.

As observed in Figure 6, secukinumab showed an overall 12-months persistence rate of 70% (95%; CI: 64–77%) and a 24-months persistence rate of 58% (95%; CI: 51–66%). Naive patients had the highest 24-months persistence rate (67%; 95% IC: 56–78%), followed by patients receiving secukinumab as a second (56%; 95% CI: 44–69%), and third or posterior (50%; 95% CI: 36–63%) line of therapy (*p* = 0.05).

Among patients withdrawing treatment, reasons for discontinuation were primary non-response (44%), loss of effectiveness (30%), adverse events (10%), loss of follow-up (5%), other medical reasons (5%), intolerance (2%), patient’s decision (1%), contraindication (1%), and death (1%).

## 4. Discussion

So far, more than 875,000 patients have been treated with secukinumab across its four approved therapeutic indications worldwide (data on file), and data pooled from RCTs and post-marketing safety surveillance support its long-term use (22, 23). The present study provides real-world experience of secukinumab in 221 patients with axSpA (AS and nr-axSpA) treated at 12 sites in Spain (Valencian Community). The study shows that secukinumab provides a rapid and sustained effectiveness up to 24 months of treatment as demonstrated by improvements in BASDAI, pain VAS, ptGA and phGA of disease, CRP levels, and enthesitis. These results confirm and expand on data obtained from secukinumab RCTs and other RWE studies (21, 24, 25). We observed that after 6 months of treatment the percentage of patients achieving at least low disease activity (BASDAI<4) increased from 9% to 48%, which was then maintained for up to 24 months. Furthermore, a quarter of patients were in remission (BASDAI<2) at month 24. These results are similar to recent data from the Spanish BIOBADASER (two of the 28 participating centers overlapped with the present study) and the European EuroSpA registries (20, 21) in which half of patients achieved a BASDAI<4 after 1 year of secukinumab treatment.

In general, BASDAI has been the most widely used activity measure both in clinical practice and research. However, European and Spanish recommendations guidelines place ASDAS-CRP as the preferred index to assess disease activity in axSpA, as it combines patient-reported outcomes and CRP (8, 26, 27). In the present study, ASDAS-CRP data was unavailable at most of the participating sites but mean CRP was within normal parameters from month 6 of treatment, reflecting an objective control of inflammation.

Patients with axSpA have reported pain as the most important feature of their disease (26), highlighting pain improvement as an essential treatment goal for patients. As with BASDAI, we observed that secukinumab improved pain VAS, as well as ptGA and phGA with a substantial early improvement—at month 6—across all three outcomes. In a prior real-world study a similar effectiveness on PtGA was seen (20) with patients attaining a 2-year mean score of 2.5 on a VAS (0–10).

When analyzing the effect of secukinumab on BASDAI per line of treatment, we observed that all patients benefited regardless of prior treatment, although the benefit was more pronounced in naïve patients and second-line patients. In naïve patients, after 6 months of treatment, mean disease activity was low (mean BASDAI<4) and this was maintained throughout the study period, underscoring an early and sustained response. In the second-line group, the benefit was evident at 6 months, but numerically smaller than in the naive group. At month 24, the mean BASDAI and change from baseline were similar in both lines of therapy, reflecting a slower but steady response. On the other hand, although patients previously treated with three or more bDMARDs showed improvements in BASDAI, the improvement was to smaller extent than the other two groups and did not reach a mean BASDAI<4. Similar to our data, a German prospective study (AQUILA) also demonstrated that naive and second-line patients were able to attain a mean BASDAI<4 at 52 weeks whereas third or posterior line patients were not (28). This observation is in line with the EuroSpA study in which the effectiveness of secukinumab was lower when the number of previous b/tsDMARD was higher (21). We also observed higher improvements in pain VAS, ptGA and phGA when secukinumab was used as first and second bDMARD, with both groups presenting a reduction of more than 50% from baseline to month 24; and, similar to BASDAI, the improvement was mostly evident during the first 6 months of treatment. In the AQUILA and BIOBADASER studies (20, 28) secukinumab also showed comparable effectiveness in naive and second-line patients, supporting the preferential use of secukinumab as an early treatment option.

Our findings also demonstrated that persistence with secukinumab was high, confirming reports from prior RWE studies (20, 21, 24, 29–31). The 24-month persistence rate (58%) was within the range (43–66%) reported by other observational studies conducted in patients with SpA in Spain (29, 31). Persistence was higher in patients receiving secukinumab as first bDMARD line than as second or posterior lines, in accordance with the abovementioned effectiveness outcomes. This trend was also observed in the study from the BIOBADASER Spanish Registry (20).

Secukinumab can be up-titrated to 300 mg (32), based on clinical response to treatment, which allows physicians flexibility in terms of dosing. In approximately 20% of the patients who started with 150 mg, the dose was increased to 300 mg; this percentage was highest in patients on a third or subsequent line of therapy. Interestingly, 20% and 23% of patients receiving secukinumab as second- or third-posterior lines, respectively, started treatment directly with the 300 mg dose, despite this strategy being off-label. Results from the clinical trial MEASURE 3 showed that even if improvements in signs and symptoms of AS patients were observed with both the 150-mg and 300-mg doses, improvements were numerically higher with the latter, especially in patients who were unresponsive to TNFi (33). Thus, uptitration in patients treated with the 150-mg dose is a viable option and can be considered before switching to another bDMARD.

Following the growing number of studies providing evidence of secukinumab efficacy and safety in the entire axSpA spectrum published in the last few years (12, 18, 20, 24, 25, 29, 31, 34–39), the recently updated ASAS/EULAR recommendations for the management of axSpA, recommend starting biologic treatment with either TNFi or IL-17i as current practice, and indicate that an IL-17i may be preferred in patients with significant psoriasis (8). Recent and ongoing RCTs designed to compare the efficacy of secukinumab as a first line bDMARD compared to TNFi (40, 41) in axSpA will shed light on whether using secukinumab as first-line bDMARD results in superior benefits compared with TNFi.

The study has several limitations that deserve to be discussed. First, given that data were gathered from a real-world setting, some study outcomes had missing data. This limitation is inherent to the observational and retrospective design of the study; however, all efforts were done to gather available data and most patients had available data for all the study variables. Second, radiographic assessments were not collected, and safety information was limited; however, the number of patients who withdrew secukinumab due to adverse events was low. Regarding effectiveness data, the lack of ASDAS-CRP assessment could also be considered limitations of the study. Third, our results can be generalized to the axSpA patients in the Valencian Community (due to the large and heterogeneous sample size and the high number of participating centers), and in all probability Spain. Further generalization of the results should be performed with caution. Future studies assessing the use of secukinumab in axSpA in the clinical setting of different regions and countries are warranted.

In conclusion, this real-world study broadens the current understanding of the real-life effectiveness and persistence of secukinumab in axSpA patients. We have shown that secukinumab rapidly improves disease activity in axSpA patients, especially in naive and second-line patients, which results in high persistence rates at 24 months of follow-up. Finally, our study shows that secukinumab use in the clinical practice is in line with the last ASAS/EULAR recommendations for the management of axSpA, which positions IL-17i as one of the first bDMARDs option as per current practice.

## Data availability statement

The raw data supporting the conclusions of this article will be made available by the authors, without undue reservation.

## Ethics statement

The study involved human participants and was reviewed and approved by Ethics Committee of the General University Hospital in Elda, Alicante, Spain. Written informed consent for participation was not required for this study in accordance with the national legislation and the institutional requirements.

## Author contributions

FS, VN-M, CC-F, IB-T, MR-V, MA-Z, MG-B, JL-G, CP-G, IM, DB-S, LY-K, AC-M, AM-C, FN-B, JS-G, and JA-S contributed to data collection. FS and JA-S designed the study, analysed the data, interpreted the data, and wrote the manuscript. All authors contributed to the article and approved the submitted version.

## Funding

The author(s) declare financial support was received for the research, authorship, and/or publication of this article. This study received funding from Novartis Farmacéutica, S.A.

## Conflict of interest

FS received honoraria as a consultant from AbbVie, Pfizer, and AstraZeneca. Grant/Research support from AbbVie, Novartis, Eli Lilly, Roche, and BMS. JS-G reports personal fees and non-financial support from Abbvie, non-financial support from BMS, personal fees from Celgene, personal fees from Janssen, non-financial support from Lilly, non-financial support from MSD, personal fees from Novartis, non-financial support from Pfizer, non-financial support from Roche, non-financial support from UCB, outside the submitted work. This study received funding from Novartis Farmacéutica, S.A. The funder had the following involvement in the study: study design, interpretation of data, the writing of this article, and the decision to submit it for publication.

## Publisher’s note

All claims expressed in this article are solely those of the authors and do not necessarily represent those of their affiliated organizations, or those of the publisher, the editors and the reviewers. Any product that may be evaluated in this article, or claim that may be made by its manufacturer, is not guaranteed or endorsed by the publisher.
