# Corrigendum: Real-world experience with secukinumab in the entire axial spondyloarthritis spectrum

**DOI:** 10.3389/fmed.2024.1462054

**Published:** 2024-09-10

**Authors:** Francisca Sivera, Victoria Núñez-Monje, Cristina Campos-Fernández, Isabel Balaguer-Trull, Montserrat Robustillo-Villarino, Marta Aguilar-Zamora, Marta Garijo-Bufort, Juan Miguel López-Gómez, Carolina Peña-González, Isabel de la Morena, Diego Bedoya-Sanchís, Liliya Yankova-Komsalova, Arantxa Conesa-Mateos, Anna Martínez-Cristóbal, Francisco Javier Navarro-Blasco, José Miguel Senabre-Gallego, Juan José Alegre-Sancho

**Affiliations:** ^1^Rheumatology Department, Hospital General Universitario de Elda, Alicante, Spain; ^2^Departament of Clinical Medicine, Universidad Miguel Hernandez, Elche, Spain; ^3^Rheumatology Department, Hospital Universitario Dr Peset, Valencia, Spain; ^4^Rheumatology Department, Hospital General Universitario, Valencia, Spain; ^5^Reumatology Unit, Internal Medicine Department, Hospital Universitario de la Plana, Villarreal, Spain; ^6^Rheumatology Department, Hospital de Sagunto, Sagunto, Spain; ^7^Rheumatology Department, Hospital Francesc de Borja, Gandía, Spain; ^8^Rheumatology Department, Hospital Clínico Universitario de Valencia, Valencia, Spain; ^9^Rheumatology Department, Hospital Marina Salud, Denia, Alicante, Spain; ^10^Rheumatology Department, Hospital General Universitari de Castelló, Castellón, Spain; ^11^Rheumatology Department, Hospital Universitario de La Ribera, Alzira, Spain; ^12^Rheumatology Department, Hospital Universitario de Elche, Elche, Alicante, Spain; ^13^Rheumatology Department, Hospital Marina Baixa, La Vila Joiosa, Spain

**Keywords:** secukinumab, effectiveness, axial spondyloarthritis, non-radiographic axial spondyloarthritis, ankylosing spondylitis, real-world evidence

In the published article, there was an error in Figure 4. In the left panel (naïve patients) there is a typo. We reported that 24 months after starting treatment with secukinumab, naïve patients showed a difference of −3.7 points. Patients had a baseline mean BASDAI score of 5.8, which decreased to 3.1 at 24 months. The reported difference is −3.7; however, the subtraction of these scores is −2.7. Similarly, we reported that 24 months after starting treatment with secukinumab, patients with third or subsequent line treatments showed a difference of −2.3 points. Patients had a baseline mean BASDAI score of 7.1, which decreased to 5.4 at 24 months. The reported difference was 2.3; however, the subtraction of these scores is −1.7.

The corrected Figure 4 and its caption appear below:

**Figure 4:**
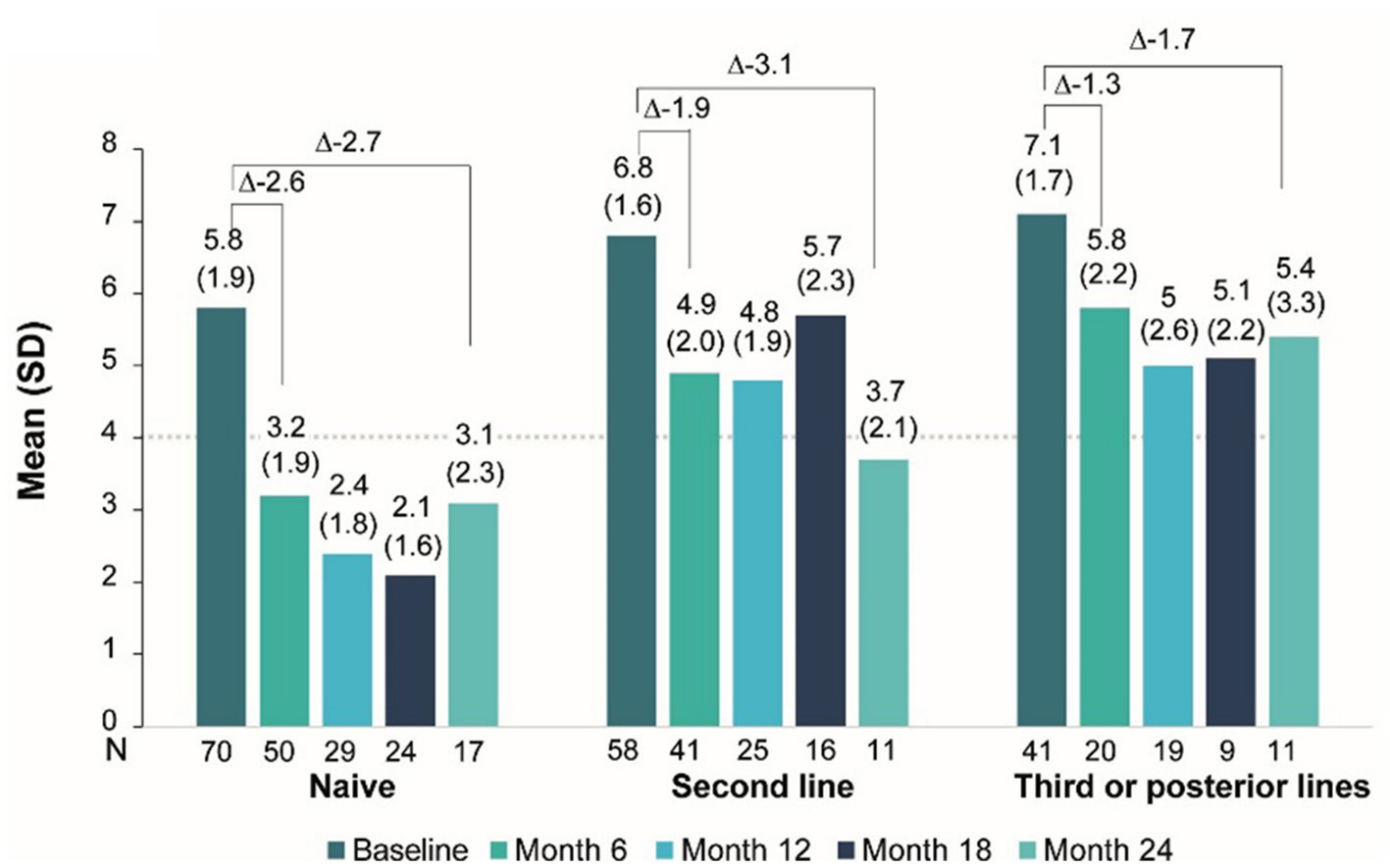
BASDAI score evolution under secukinumab treatment by line of treatment.

In the published article, there was an error. The typo mentioned in Figure 4, was also included in the text.

A correction has been made to **Abstract (Results)**. This sentence previously stated:

“The greatest improvement in BASDAI was observed in naïve patients (month 6: −2.6; month 24: −3.7), followed by second-line (month 6: −1.9; month 24: −3.1) and ≥thirdline (month 6: −1.3; month 24: −2.3) patients.”

The corrected sentence appears below:

“Improvements in BASDAI were observed across all treatment lines: in naïve patients (month 6: −2.6; month 24: −2.7), in second-line (month 6: −1.9; month 24: −3.1), and in patients on ≥third lines (month 6: −1.3; month 24: −1.7).”

A correction has been made to **3. Results, 3.3.2. Per treatment line**. This sentence previously stated:

“As shown in Figure 4, even though mean BASDAI improved across all lines of treatment, naïve patients showed the greatest BASDAI improvement, whereas patients on third or subsequent lines of therapy showed the least improvement.”

The corrected sentence appears below:

“As shown in Figure 4, even though mean BASDAI improved across all lines of treatment, naïve patients and second-line patients showed the greatest BASDAI improvement, whereas patients on third or subsequent lines of therapy showed the least improvement.”

A correction has been made to **4. Discussion**. This paragraph previously stated:

“When analyzing the effect of secukinumab on BASDAI per line of treatment, we observed that naive patients benefited the most from secukinumab treatment. After 6 months of treatment mean disease activity was low (mean BASDAI < 4) and this was maintained throughout the study period, underscoring an early and sustained response. In the second-line group, the benefit was evident at 6 months, but numerically smaller than in the naive group. At month 24, the mean BASDAI and change from baseline were similar in both lines of therapy, reflecting a slower but steady response. On the other hand, although patients previously treated with three or more bDMARDs showed improvements in BASDAI, the improvement was to smaller extent than the other two groups and did not reach a mean BASDAI < 4.”

The corrected sentence appears below:

“When analyzing the effect of secukinumab on BASDAI per line of treatment, we observed that all patients benefited regardless of prior treatment, although the benefit was more pronounced in naïve patients and second-line patients. In naïve patients, after 6 months of treatment, mean disease activity was low (mean BASDAI < 4) and this was maintained throughout the study period, underscoring an early and sustained response. In the second-line group, the benefit was evident at 6 months, but numerically smaller than in the naive group. At month 24, the mean BASDAI and change from baseline were similar in both lines of therapy, reflecting a slower but steady response. On the other hand, although patients previously treated with three or more bDMARDs showed improvements in BASDAI, the improvement was to smaller extent than the other two groups and did not reach a mean BASDAI < 4.”

In the published article, there was an error in the Funding statement. This statement previously stated:

“This study has been sponsored by Novartis Farmacéutica, S.A. The funder was not involved in the study design, collection, analysis, interpretation of data, the writing of this article, or the decision to submit it for publication. All authors declare no other competing interests.”

The correct Funding statement appears below:

