# Supplementary material for: Real-world experience with secukinumab in the entire axial spondyloarthritis spectrum
Source: Front Med (Lausanne). 2023 May 11;10:1156557. doi: 10.3389/fmed.2023.1156557 (PMC10213893; doi:10.3389/fmed.2023.1156557)
Supplement: Supplementary file 1 [file Table_1.DOCX]

Supplementary Material

Real-world experience with secukinumab in the entire axial spondyloarthritis spectrum

Francisca Sivera ^1^†, Victoria Núñez Monje^2^, Cristina Campos-Fernández^3^, Isabel Balaguer-Trull^3^, Montserrat Robustillo Villarino^4^, Marta Aguilar-Zamora^5^, Marta Garijo-Bufort^5^, Juan Miguel López-Gómez^1^, Carolina Peña-González ^6^, Isabel de la Morena^7^, Diego Bedoya-Sanchís^7^, Liliya Yankova-Komsalova^8^, Arantxa Conesa-Mateos^9^, Anna Martinez-Cristóbal^10^, Francisco Javier Navarro-Blasco^11^, Jose Miguel Senabre-Gallego^12^, José Alegre-Sancho^2^†*

^1^ Rheumatology Department, Hospital General Universitario de Elda, Alicante, Spain

^2^ Rheumatology Department, Hospital Universitario Dr Peset, Valencia, Spain

^3^ Rheumatology Department, Hospital General Universitario, Valencia, Spain

^4^ Internal Medicine Department, Reumatology unit, Hospital Universitario de la Plana, Villareal, Spain

^5^ Rheumatology Department, Hospital de Sagunto, Sagunto, Valencia, Spain

^6^ Rheumatology Department, Hospital Francesc de Borja, Gandía, Valencia, Spain

^7^ Rheumatology Department, Hospital Clínico Universitario de Valencia, Valencia, Spain

^8^ Rheumatology Department, Hospital Marina Salud, Denia, Alicante, Spain

^9^ Rheumatology Department, Hospital General Universitari de Castelló, Castellón, Spain

^10^ Rheumatology Department, Hospital Universitario de La Ribera, Alzira, Valencia, Spain

^11^ Rheumatology Department, Hospital Universitario de Elche, Elche, Alicante, Spain

^12^ Rheumatology Department, Hospital Marina Baixa, La Vila Joiosa, Spain

†these authors contributed equally to this work

*** Correspondence:**Juan José Alegre-Sancho, Hospital Dr. Peset, Av. de Gaspar Aguilar, 90, 46017 València, Valencia. Email: [alegre_juasan@gva.es](mailto:alegre_juasan@gva.es)

# Supplementary Tables

## Supplementary Table 1 Patient characteristics and secukinumab treatment by diagnosis

|  | **AS (n=181)** | **Nr-axSpA (n=39)** |
| --- | --- | --- |
| **Baseline demographic and clinical characteristics** |  |  |
| Gender (male), n (%) [N] | 129 (71) [181] | 23 (59) [39] |
| Ethnicity (Caucasian), n (%) [N] | 170 (93) [181] | 34 (87) [39] |
| Age, mean (SD) [N] | 48.3 (12) [181] | 39.4 (9.6) [39] |
| BMI (kg/m^2^), mean (SD) [N] | 27.6 (5) [103] | 27.1 (6.4) [23] |
| Obese (BMI ≥30), n (%) [N] | 32 (31.1) [103] | 8 (34.8) [23] |
| Current smokers, n (%) [N] | 55 (37) [148] | 9 (27) [33] |
| Age at symptoms initiation, mean (SD) [N] | 35.7 (12.2) [145] | 35.5 (9.2) [33] |
| Age at diagnosis, mean (SD) [N] | 39.9 (12.1) [171] | 36.7 (9.6) [38] |
| Years from first symptoms to diagnosis, mean (SD) [N] | 4.5 (6.4) [145] | 3 (3.3) [33] |
| Peripheral arthritis, n (%) [N] | 47 (26) [181] | 15 (39) [39] |
| Enthesitis (yes), n (%) [N] | 22 (15) [144] | 7 (24) [29] |
| Number at baseline assessment, mean (SD) [N] | 2.5 (2.1) [19] | 2.4 (1.1) [7] |
| Dactylitis (yes), n (%) [N] | 0 (0) [146] | 2 (7) [29] |
| Skin manifestation (yes), n (%) [N] | 17 (10) [166] | 1 (3) [35] |
| Nail manifestation (yes), n (%) [N] | 2 (1) [166] | 0 (0) [35] |
| Uveitis (yes), n (%) [N] | 10 (6) [167] | 2 (6) [35] |
| Prior number of flares, mean (SD) [N] | 2.1 (0.9) [9] | 1 (1) [1] |
| BASDAI, mean (SD) [N] | 6.4 (1.9) [139] | 6.7 (1.6) [30] |
| Pain VAS, mean (SD) [N] | 66.6 (23.9) [113] | 69.4 (18.3) [25] |
| PtGA, mean (SD) [N] | 65.4 (23.8) [84] | 65.9 (23.6) [16] |
| PhGA, mean (SD) [N] | 54.7 (22.5) [71] | 59.7 (21) [17] |
| CRP (mg/dl), mean (SD) [N] | 6.9 (14) [169] | 5.2 (6.5) [37] |
| ESR (mm/h), mean (SD) [N] | 21.3 (20.7) [161] | 18.9 (19.9) [37] |
| HLA-B27 (positive), n (%) [N] | 133 (77) [173] | 22 (58) [38] |
| Prior anti-TNFα, n (%) [N] | 113 (82) [138] | 23 (72) [32] |
| Comorbidities (any), n (%) [N] | 91 (50) [181] | 8 (21) [39] |
| Hypertension (yes), n (%) [N] | 46 (51) [91] | 3 (38) [8] |
| Dyslipidemia (yes), n (%) [N] | 37 (41) [91] | 1 (13) [8] |
| Depression (yes), n (%) [N] | 24 (26) [91] | 3 (38) [8] |
| Diabetes (yes), n (%) [N] | 12 (13) [91] | 0 (0) [8] |
| Tuberculosis/latent tuberculosis infection (yes), n (%) [N] | 23 (25) [91] | 1 (13) [8] |
| Fatty liver disease (yes), n (%) [N] | 8 (9) [91] | 4 (50) [8] |
| Cardiovascular events (yes), n (%) [N] | 10 (11) [91] | 0 (0) [8] |
| Neoplasms (yes), n (%) [N] | 11 (12) [91] | 0 (0) [8] |
| **Secukinumab treatment** |  |  |
| Years from first symptoms to secukinumab initiation, mean (SD [N] | 12.3 (9.3) [145] | 5.4 (3.9) [33] |
| Years from diagnosis to secukinumab initiation, mean (SD [N] | 8.4 (8) [171] | 2.4 (2) [38] |
| Initial dose: 150 mg, n (%) [N] | 154 (85) [181] | 36 (92) [39] |
| Monotherapy, n (%) [N] | 143 (80) [181] | 30 (77) [39] |
| Duration of secukinumab (months), mean (SD) [N] | 20.9 (16) [181] | 20.9 (15) [39] |

One patient with axSpA did not have data regarding AS or nr-axSpA diagnosis; therefore, data from this patient has not been included here.

## Supplementary Table 2 Prior treatment

|  | **AS** | **Nr-axSpA** | **axSpA** |
| --- | --- | --- | --- |
| **Treatment** |  |  |  |
| Prior DMARD (yes), n (%) [N] | 138 (76) [181] | 32 (82) [39] | 171 (77) [221] |
| Biologic (anti-TNFα) | 113 (82) [138] | 23 (72) [32] | 137 (80) [171] |
| Adalimumab | 70 (62) [113] | 12 (52) [23] | 83 (61) [171] |
| Etanercept | 53 (47) [113] | 8 (35) [23] | 62 (45) [137] |
| Golimumab | 33 (29) [113] | 5 (22) [23] | 38 (28) [137] |
| Certolizumab | 18 (16) [113] | 6 (26) [23] | 24 (18) [137] |
| Infliximab | 16 (14) [113] | 2 (9) [23] | 18 (13) [137] |
| Conventional synthetic | 85 (62) [138] | 21 (66) [32] | 107 (63) [171] |
| Metotrexate | 55 (65) [85] | 15 (71) [21] | 71 (66) [107] |
| Leflunomide | 4 (5) [85] | 2 (10) [21] | 6 (6) [107] |
| Sulfasalazine | 57 (67) [85] | 13 (62) [21] | 70 (65) [107] |
| Other | 2 (2) [85] | 0 | 2 (2) [107] |

*Note:* Valid percentages (i.e. percentage of patients with data in the variable) are showed here.

One patient could had more than one previous treatment.

## Supplementary Table 3 Evolution of BASDAI

| **BASDAI** | **Baseline** | **Month 6** | **Month 12** | **Month 18** | **Month 24** |
| --- | --- | --- | --- | --- | --- |
| Mean (SD) | 6.4 (1.8) | 4.3 (2.2) | 3.9 (2.4) | 3.8 (2.6) | 3.9 (2.6) |
| N | 170 | 111 | 73 | 49 | 39 |

## Supplementary Table 4 Changes in pain VAS, ptGA and phGA after secukinumab initiation per treatment line

| **Secukinumab** | **Baseline** | **Month 6** | **Month 12** | **Month 18** | **Month 24** |
| --- | --- | --- | --- | --- | --- |
|  | **Mean (SD) [N]** | | | | |
| **Pain VAS** | | | | | |
| First line | 66.2 (19.4) [63] | 36.0 (23.1) [42] | 27.1 (24.7) [21] | 23.2 (15.9) [19] | 30.0 (28.9) [17] |
| Second line | 64.5 (26.9) [45] | 47.1 (27.1) [37] | 39.8 (28.9) [20] | 54.4 (16.4) [8] | 30.1 (24.2) [11] |
| ≥Third line | 70.7 (25.6) [30] | 52.8 (28.6) [18] | 50.1 (32.4) [12] | 60.0 (8.2) [4] | 51.5 (35.8) [8] |
| **PtGA** | | | | | |
| First line | 66.2 (18.9) [48] | 36.4 (24.6) [32] | 29.3 (26.2) [15] | 21.5 (16.5) [14] | 29.6 (29.9) [12] |
| Second line | 61.8 (27.8) [29] | 37.4 (26.4) [27] | 32.9 (29.2) [13] | 49.2 (30.4) [6] | 30.2 (27.9) [9] |
| ≥Third line | 66.0 (29.7) [23] | 53.0 (29.2) [13] | 43.8 (28.4) [8] | 60.0 (14.1) [2] | 44.0 (38.0) [6] |
| **PhGA** | | | | | |
| First line | 58.5 (18.3) [46] | 25.3 (17.5) [30] | 29.7 (25.9) [16] | 23.1 (21.8) [16] | 25.5 (23.9) [13] |
| Second line | 50.7 (26.2) [26] | 29.9 (19.6) [21] | 22.1 (15.8) [10] | 37.2 (25.5) [5] | 15.5 (14.0) [9] |
| ≥Third line | 52.5 (27.2) [16] | 43.0 (28.5) [11] | 42.9 (27.5) [7] | 20.0 (-) [1] | 36.0 (37.8) [5] |
